# Anthropomorphic and Physical Fitness Characteristics of United States Air Force Basic Military Training: Special Warfare Versus Nonspecial Warfare Recruits, Fiscal Year 2019-2023

**DOI:** 10.7759/cureus.63391

**Published:** 2024-06-28

**Authors:** John D Mata, Amanda L Patrick, Juste N Tchandja, Lauren E Haydu, Kathleen K Hogan, Korey B Kasper, Steven D Trigg, Cody R Butler

**Affiliations:** 1 Special Warfare Human Performance Squadron, United States Air Force, San Antonio, USA; 2 Biostatistics, Mayo Clinic, Jacksonville, USA; 3 Trainee Health, 559th Medical Group, San Antonio, USA

**Keywords:** covid-19, special operations, air force, physical fitness training, exercise sciences, active-duty military personnel

## Abstract

Introduction: Each year, thousands of individuals enlist in the Department of the Air Force (DAF), with some seeking to become DAF Special Warfare (SW) candidates. This study aimed to compare the anthropomorphic and physical fitness characteristics between these groups during fiscal years (FYs) 2019-2023.

Methods: The sample includes male candidates below the age of 30 who attended the DAF basic military training (BMT) from FY2019 to 2023 (N = 119,415). Initial physical fitness testing was conducted during week 1 of BMT. Physical fitness results, height, weight, and body mass index (BMI) were compared between the two cohorts. A two-way analysis of variance was performed to analyze the effects of group (SW and non-SW) and FY on mean anthropomorphic and physical fitness test variables. Dependent variables were evaluated for homogeneity of variance using Levene’s test and for normality using the Shapiro-Wilk test. The Tukey-Kramer test was employed for post hoc analyses with a threshold for statistical significance of α < 0.05.

Results: The cohort of SW recruits displayed superior physical fitness results across all FYs (p < 0.001) with the exception of FY2021. They were significantly taller and heavier, and had a higher BMI when compared to non-SW DAF BMT recruits (p < 0.001). Mean values for maximum push-ups and sit-ups for SW recruits were significantly lower in FY2021 (p < 0.001) and not significantly different from non-SW recruits. Additionally, run times for both SW- and non-SW-bound recruits during FY2022 and FY2023 were significantly slower than previous years.

Conclusions: These findings can be used to establish a baseline for anthropometric and physical fitness profiles of incoming SW and non-SW DAF BMT recruits that may inform clinicians, human performance professionals, and military training leaders with information necessary to guide future research and physical fitness policy.

## Introduction

Each year, approximately 35,000 individuals enlist in the Department of the Air Force (DAF) [1]. The first stop along their journey is Joint Base San Antonio-Lackland, where they will undergo 7.5 weeks of basic military training (BMT) before earning the title of "Airman." The training in this course of initial entry consists of an introduction to the Air Force lifestyle, history, culture, military drill and ceremony, personal appearance and conduct, standards of dress, followership, leadership, a simulated deployed environment, basic nutrition, and physical fitness. During a prospective airman’s development, there is a continual emphasis placed on the Air Force's core values: (1) “integrity first,” (2) “service before self,” and (3) “excellence in all we do.” Upon successful completion of BMT requirements, which include passing an end-of-course academic exam and a physical fitness assessment (PFA), these airmen will receive their formal orders to proceed to their technical training assignments and begin learning their individually assigned career specialty at the respective technical training school.

The BMT PFA is completed at three separate time points over the course of BMT. The first assessment occurs during the first week of training (1WOT). Its purpose is to inform trainees and their military training instructors on how much improvement is necessary to achieve a passing score by the end of BMT, which is required to graduate. This PFA consists of three subtests: maximum push-ups (MPU) in 60 seconds, maximum sit-ups (MSU) in 60 seconds, and a timed 1.5-mile run (RUN). In addition, trainee height (HT) and weight (WT) are measured, and subsequent body mass index (BMI) calculations are conducted in 1WOT. The scoring criteria for these events are classified into male and female standards across separate age categories. Of note, BMT has separate standards from the active-duty component of the Force, which are outlined in the 737th Training Group (TRG) Standard Operating Procedures (SOP) 36-2905, dated May 2022, and DAF Manual 36-2905, respectively.

In fiscal years 2019-2023 (FY19 and FY23, respectively), a total of 171,913 recruits entered DAF BMT. Of those recruits, 3,900 (2.3%) sought the arduous path to become DAF Special Warfare (AFSPECWAR) candidates and hopefully move into one of four highly specialized enlisted career fields: pararescue (PJ), combat control (CCT), special reconnaissance (SR), or tactical air control party (TACP). Special Warfare (SW) recruits complete BMT within their own groups (flights) but must still complete all training and educational requirements as those not bound for AFSPECWAR. The AFSPECWAR BMT flights participate in a different physical fitness program intended to prepare them for the physically demanding training pipeline, which can last for more than two years beyond BMT. Airmen who successfully complete AFSPECWAR training requirements move into these specialties. They are expert ground combat personnel who specialize in delivering precision air power, command and control, emergency personnel recovery, trauma and field medical care, and multidomain reconnaissance across a full spectrum of military operations in any environmental conditions [2]. Exceptional physical fitness is vital for an AFSPECWAR operator to complete their unique mission [3]. Integrity, drive, problem-solving, stress tolerance, trainability, communication skills, and teamwork are also essential. These character traits are deemed vital to completing their unique mission. Each of these qualities is developed and tested during AFSPECWAR training.

As of the time of this study, there have been no formal comparisons of the anthropometric profiles (HT and WT) and physical fitness performances, as measured by the PFA, between SW recruits set to embark into PJ, CCT, SR, or TACP and traditional DAF (non-SW) recruits intended for all other career fields. The main purpose of this study is to compare the initial physical characteristics of these two groups when reporting to BMT during FY19-FY23. In addition, physical fitness testing comparisons were conducted to identify potential significant relationships by intended career type between the two cohorts. These findings can be used to establish a baseline for anthropometric and physical fitness profiles of incoming SW and non-SW DAF BMT recruits that may inform clinicians, human performance professionals, and military training leaders with information necessary to guide future research and physical fitness policy.

## Materials and methods

Data sources, setting, and participants

**Figure 1:**
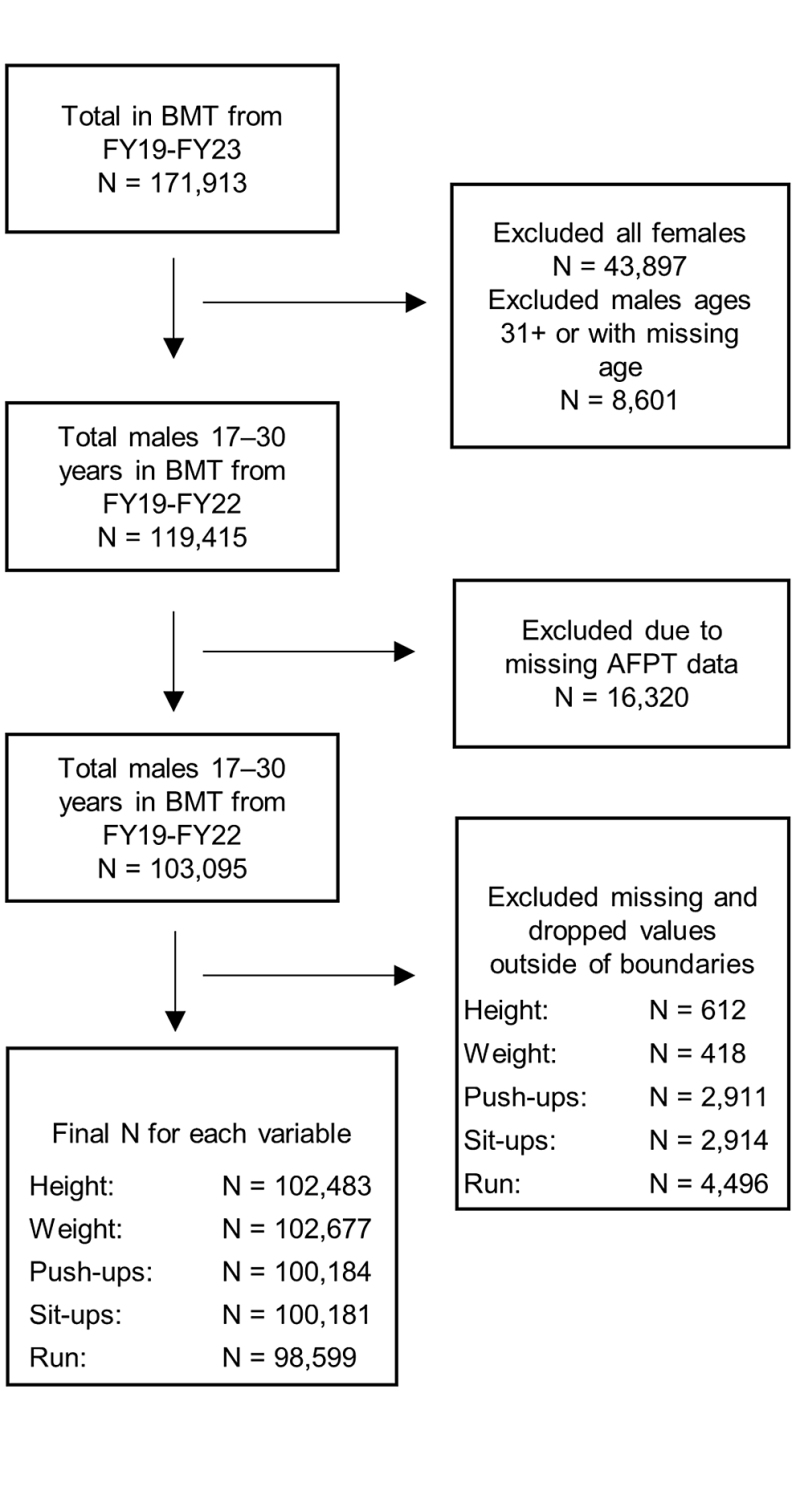
CONSORT diagram for PFA analysis BMT: basic military training; AFPT: Armed Forces Prerecruitment Training; CONSORT: Consolidated Standards of Reporting Trials; PFA: physical fitness assessment

The cohort includes all candidates who attended DAF BMT from FY19 to FY23. Females (N = 43,897) were excluded from this analysis due to the ample evidence suggesting distinct physiological and physical performance differences between men and women and the paucity of women vectoring into AFPSECWAR training. Additionally, all DAF recruits above 30 years old were excluded from this comparison (N = 8,601). After all females and candidates above the age of 30 were excluded (N = 52,498), there were 119,415 males between the ages of 17 and 30 years to be compared (N = 3,604 SW and 115,811 non-SW) within anthropomorphic analyses. An additional 16,320 males between the ages of 17 and 30 were excluded from all fitness comparisons due to missing records, resulting in a cohort of 103,095 (N = 3,323 SW and 99,772 non-SW) for all fitness evaluations (Figure 1).

The PFAs were conducted before participation within either the SW BMT or traditional BMT physical fitness programs in order to make this comparison. All initial PFA testing was conducted during week 1 of BMT, before participation in any structured physical fitness program at BMT. Recruits’ PFA performance data were collected from the Electronic Basic Training Management System. Demographic data were collected from the Air Education and Training Command Technical Training Management System with the intended Air Force Specialty Code (AFSC) upon enlistment utilized to identify the BMT group (SW or non-SW). Those with a corresponding SW AFSC were assigned "SW = Yes," and all others were designated “SW = No.” Existing HT and WT (WT) values were used to calculate BMI using the following formula: BMI = (HT/WT^2^) × 703.

Development of data exclusion criteria

Anthropomorphic values outside of normal ranges that were identified after initial data quality evaluation were removed. Abnormal HT and WT ranges were defined by reviewing official BMT minimum and maximum standards for those variables, as defined in the 737th TRG SOP 36-2905 and DAF Body Composition Program Policy Memorandum. Individuals who were outside of a combined HT and WT standard (Table 1) were assumed to be either erroneously input or required an official exemption to policy (ETP). Persons requiring ETP who were able to begin BMT were thus evaluated on a case-by-case basis by BMT staff. The authors excluded these entries to preserve the integrity of the data, as the authors did not have access to the documentation and rationale for these individual decisions. The accepted ranges for PFA physical performance variables were deduced by examining physiological performances across similar populations, world, national, and state records for the events in question, and the distribution of our current data set. Those values that were outside of the identified and accepted normal ranges for physical fitness or anthropometric variables received “n/a” in place of the erroneous entry in all comparisons. Due to variability within each data subset, those counts specific to each analysis will be reflected in each table below.

**Table 1:** Accepted data ranges for PFA NA: not applicable; MSU: maximum sit-ups; MPU: maximum push-ups; PFA: physical fitness assessment

Variable	Minimum	Maximum
Height (in.)	58	80
Weight (lbs.)	84	260
1.5-mile run (minutes)	6	NA
Sit-ups (MSU; number of repetitions)	0	200
Push-ups (MPU; number of repetitions)	0	200

Statistical methods

Independent t tests and chi-square tests were used to generate tables in this analysis. All analyses and figures were performed and created with the Statistical Analysis Software (SAS) version 9.4 (SAS Institute Inc., Cary, NC). A two-way analysis of variance (ANOVA) was performed to analyze the effect of independent variables (group [SW and non-SW] and FY) on mean values of dependent variables (1.5-mile run [RUN], push-up [MPU], sit-up [MSU], HT, WT, and BMI). Dependent variables were tested for homogeneity of variance using Levene’s test and for normality using the Shapiro-Wilk test. The Tukey-Kramer test was employed for post hoc analyses with a threshold for statistical significance of α < 0.05.

## Results

There were 103,095 candidates in the analysis who attended BMT from FY19 to FY23, and 3.2% (N = 3,323) intended to pursue AFSPECWAR career fields (SW cohort). After excluding individuals who were more than 31 years old, the mean age of SW (23.4 years, SD = 3.0) was higher than the non-SW cohort (22.7 years, SD = 2.8 years; p < 0.001; Table 2). The mean HT of SW (70.0 in., SD = 2.7 in.) was negligibly higher than the non-SW cohort (69.6 in., SD = 2.9 in.; p < 0.001; Table 2). The mean WT of SW (169.5 lbs., SD = 19.7 lbs.) was statistically higher than the non-SW cohort (165.3 lbs., SD = 23.5 lbs.; p < 0.001; Table 2). The mean BMI of SW (24.3, SD = 2.3) was statistically higher than the non-SW cohort (24.0, SD = 2.9; p < 0.001; Table 2).

**Table 2:** Baseline anthropometric and fitness values by SW-status for males 17–30 years, during FY19-FY23 All continuous data are compared with independent t tests. Categorical data are compared with Pearson chi-square tests *Recruits admitted to BMT are reduced due to social distancing mitigation strategies imposed in response to the COVID-19 pandemic BMI: body mass index; SW: special warfare; FY: fiscal year; BMT: basic military training

Factor	Total N = 103,095			SW: N = 3,323			Non-SW: N = 99,772			P value	Effect size	Magnitude
	N	Mean	SD	N	SW	SD	N	Non-SW	SD			
Age (years)	103,095	22.75	2.81	3,323	23.35	3.04	99,772	22.73	2.8	<0.001	-0.22	Small
Height (in.)	102,483	69.64	2.92	3.313	70.03	2.67	99,170	69.62	2.93	<0.001	-0.14	Negligible
Weight (lbs.)	102,677	165.43	23.42	3,316	169.53	19.71	99,361	165.29	23.52	<0.001	-0.18	Negligible
BMI	102,428	23.97	2.9	3,310	24.29	2.31	99,118	23.96	2.92	<0.001	-0.11	Negligible
1.5-mile run (minutes:seconds)	98,599	13:14	5:34	3,037	10:33	5:32	95,562	13:20	5:32	<0.001	0.50	Moderate
Sit-ups	100,181	40.86	13.19	3,276	50.65	16.44	96,905	40.53	12.93	<0.001	-0.78	Moderate
Push-ups	100,184	41.43	15.48	3,275	53.49	18.28	96,909	41.02	15.22	<0.001	-0.81	Large
FY	Count		Percent	Count		Percent	Count		Percent	P value	Effect size	Magnitude
2023	21,520		20.9%	702		21.1%	20,818		20.9%	<0.001	0.04	Weak
2022	17,093		16.6%	777		23.4%	16,316		16.4%			
2021	24,415		23.7%	793		23.9%	23,622		23.7%			
2020	14,339*		13.9%	413		12.4%	13,926		14.0%			
2019	25,728		25.0%	638		19.2%	25,090		25.1%			

Anthropometric characteristics

**Figure 2:**
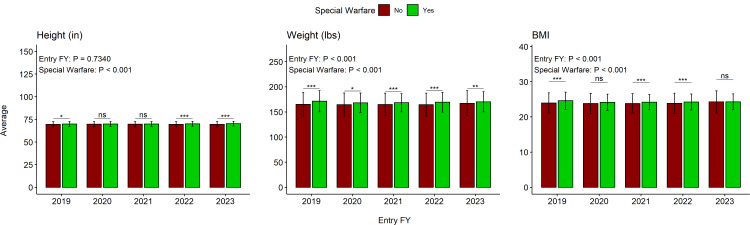
Pairwise anthropometric comparisons between SW and non-SW recruits Bar charts between anthropometric measures are not meant to be compared; y-axes measure mean value, and each error bar represents the SD. Entry FY p value and SW p value represent the main effects included in two-way ANOVA FY: fiscal year; ns: not significant; BMI: body mass index; SW: special warfare; ANOVA: analysis of variance *<0.01; **<0.01; ***<0.001

The SW cohort exhibited significantly greater weight, height, and BMI in comparison to non-SW candidates (p < 0.001; Table 2). When comparing FYs, there were significant differences for WT (p < 0.001) and BMI (p < 0.001) but not for HT (p = 0.740) between the groups (Figure 2). The differences between the SW and non-SW groups were consistent across FYs, as indicated by the lack of significant interaction of the two independent variables for HT (F = 1.66, p = 0.156) and WT (F =1.90, p = 0.107). The interaction analysis revealed that there was a significant interaction between SW and FY for BMI (F = 4.05, p = 0.003). The SW cohort had a slightly higher BMI for FY19, FY21, and FY22 (Figure 2). All FY23 candidates had significantly greater WT compared with FY20 and FY21 candidates (p = 0.029 and 0.024, respectively). All candidates in FY19 had slightly higher BMI than those in FY20 and FY21 (p = 0.0326 and 0.0193, respectively), and all candidates in FY23 had slightly higher BMI than those in FY20 and FY21 (p = 0.0293 and 0.0161, respectively).

Physical fitness comparison

**Figure 3:**
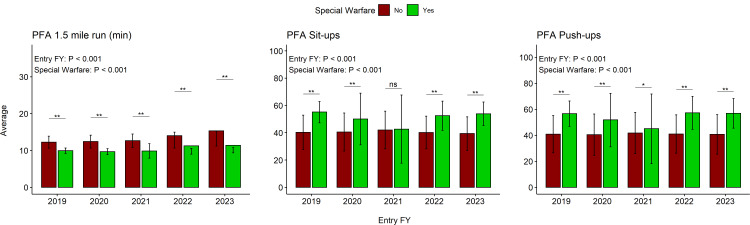
Pairwise PFA comparisons between SW and non-SW recruits Bar charts between PFA events are not meant to be compared; y-axes measure mean value, and each error bar represents the SD. Entry FY p value and SW p value represent the main effects included in two-way ANOVA PFA: physical fitness assessment; FY: fiscal year; ns: not significant; SW: special warfare; ANOVA: analysis of variance *<0.01; **<0.001

SW candidates were, on average, 2 minutes and 47 seconds faster during RUN than non-SW candidates (p < 0.001; Table 3). Two-way ANOVA revealed that RUN times were significantly higher for FY22 compared with previous FYs (i.e., FY19, FY20, and FY21; Figure 3), for RUN in FY23 compared with previous FYs (i.e., FY19-FY22), and for SW compared with non-SW. Interaction analysis revealed there was a significant interaction between FY and SW status (p < 0.001).

**Table 3:** Two-way ANOVA results comparing SW status and entry FY DF: degrees of freedom; SS: sums of squares; MS: mean squares; SW: special warfare; FY: fiscal year; BMI: body mass index; ANOVA: analysis of variance

Factor	Source	DF	Type III SS	MS	F value	Pr > F
Height	SW status	1	500.56	500.56	58.70	<0.001
	Entry FY	4	16.88	4.22	0.49	0.740
	Interaction	4	56.59	14.15	1.66	0.156
Weight	SW status	1	52,984.06	52,984.06	96.87	<0.001
	Entry FY	4	11,072.93	2,768.23	5.06	<0.001
	Interaction	4	4,157.37	1,039.34	1.90	0.107
BMI	SW status	1	343.65	343.65	40.89	<0.001
	Entry FY	4	211.95	52.99	6.31	<0.001
	Interaction	4	136.16	34.04	4.05	0.003
1.5-mile run time	SW status	1	23,608.46	23,608.46	805.45	<0.001
	Entry FY	4	10,224.64	2,556.16	87.21	<0.001
	Interaction	4	982.16	245.54	8.38	<0.001
Sit-ups	SW status	1	321,792.31	321,792.31	1,902.71	<0.001
	Entry FY	4	50,433.24	12,608.31	74.55	<0.001
	Interaction	4	103,022.35	25,755.59	152.29	<0.001
Push-ups	SW status	1	475,426.99	475,426.99	2,032.28	<0.001
	Entry FY	4	70,412.63	17,603.16	75.25	<0.001
	Interaction	4	92,284.38	23,071.10	98.62	<0.001

The cohort of SW candidates completed 10 more MSU, on average, than non-SW candidates (p < 0.001; Table 3). The SW candidates completed significantly more MSU for all FYs except for FY21. There were significant differences when comparing FYs except for the comparison of FY20 and FY22, FY19 and FY23, and FY22 and FY23. Overall, SW candidates performed better during MSU testing in FY19 and FY23 than in all other FYs and completed the least MSU in FY21. For non-SW recruits, MSU were consistent for all FYs. SW and non-SW MSU were significantly different for all FYs except for FY21, indicated by a significant interaction between FY and SW status (p < 0.001).

The cohort of SW candidates completed significantly more MSU than non-SW on average (p < 0.001; Table 3). The number of MPU was consistent when comparing all FYs for the non-SW cohort. Interaction analysis revealed there was a significant interaction between FY and SW status for MPU (p < 0.001). The SW cohort completed a significantly greater number of MPU than the non-SW cohort in all FYs except FY21.

## Discussion

The observed physical performance disparity observed between the groups, favoring the SW group, was expected by the authors. These data were investigated to provide a formal comparison of two distinct groups who entered DAF BMT at the same point in their military careers but had divergent physical fitness expectations. This information may provide military physical fitness leaders and future DAF BMT recruits, regardless of career field designation, with useful information to formulate policies or expectations relevant to DAF enlistment. In these analyses, SW-bound recruits ran faster 1.5-mile times across all FYs (p < 0.001), did more MPU and MSU in all FYs (p < 0.001) except FY21, were significantly taller (p < 0.001), heavier (p < 0.001), and exhibited greater BMI (p < 0.001) across all FYs except FY23 when compared to non-SW bound DAF BMT recruits. This difference is consistent with reports of physical profiles and physical fitness in similar military cohorts, especially when categorizing recruits moving into combat-arm professions or Special Operations Selection Courses versus those enlisting in noncombat support specialties [3,4]. Previous literature has reported that recruits who have attended Special Forces Assessment and Selection or other combat-related training courses previously performed several years of physical fitness training before attending the course. Most SW recruits participate in physical fitness training with developers before BMT and are acutely aware of the high physical capacity that is required to succeed in their chosen career fields. Thus, they have routinely displayed increased strength, endurance, and total muscle mass compared to their fellow recruits who did not plan to attempt these courses [4,5].

Though not analyzed or qualified in this investigation, it is expected that SW-bound recruits have a longer specific training history, are likely more motivated to complete physical training than their non-SW counterparts, and have greater inherent expectations for performance due to the desired trajectory of their military careers. All SW-bound recruits must complete and pass an initial fitness test with Air Force Recruiting Services before arriving at BMT and once again before they are allowed into the formal SW training pipeline [6]. Individuals must achieve a 1.5-mile run in less than 10:20 (minutes:seconds), 40 push-ups, and 50 sit-ups, along with swim testing. This preattendance testing and emphasis on fitness may have highly likely further influenced the superior physical fitness profiles observed in this investigation. In contrast, non-SW careers do not have a preaccession fitness test requirement before shipping to BMT.

Mean values for MPU and MSU within the cohort of SW recruits were significantly lower in FY21 and not significantly different from non-SW recruits. Additionally, run times during FY22 were significantly slower (p < 0.001) than all prior years for both SW- and non-SW-bound recruits. These observed relationships for MPU and MSU in FY21 and run times in FY22 do not follow those observed in all other years within this investigation. Inferior physical fitness, when compared to historical norms, has been observed after the COVID-19 pandemic in samples of children, adults, athletes, and military populations in the United States and globally [7-9]. During this time, the DAF approved numerous organizational policies aimed at maintaining airman production numbers despite social distancing efforts, infection-control procedures, and decreased physical performance qualities in recruits who attended BMT in response to COVID-19. These physical fitness deficits, as previously discussed, have been attributed to a decline in physical activity and organized sports in children and adolescents, especially those with a lower socioeconomic background observed during the pandemic [10].

It has been reported that when such key opportunities for social connection are reduced or eliminated, feelings of loneliness and isolation increase in high school and college-aged persons [11,12]. Moreover, young Americans who live in single-parent households are more likely to enlist in the military, and those individuals exhibit feelings of social isolation [13]. As physical activity and organized sports participation decreased within government and organizational COVID-19 social-distancing efforts within FY20-FY22, those who would have been interested in military enlistment were affected to a great degree on several socioecological fronts. The authors believe this relationship can be considered a contributory combination of factors for the observed decreased aerobic and muscular endurance performances displayed within both groups during FY21-FY22.

Limitations

A major limitation of this investigation is the total amount of missing data present within the datasets, both within dependent and independent variables. The discrepancy in missing data proportions by group and the fact that different data were missing within varied dependent variable categories between the unique individuals proved a challenge to consistent comparisons within each section of this manuscript. The impact of these missing data was not easily mitigated due to the post hoc nature of this inquiry, and without the existence of detailed annotation, routine data quality reviews, or manual logs of missing data sources, the authors were forced to exclude large amounts of data, which could have possibly been prevented through more detailed documentation and data stewardship.

Another limitation of this study was the worldwide impact of the COVID-19 pandemic, which occurred during the times of observation. As outlined in the 737th Training Support Squadron, Human Performance Directorate Published in 2021 [14], DAF BMT revamped its physical training program multiple times during the pandemic due to inherent challenges to fitness outcomes in the face of physical/social distancing mandates and reduction of BMT training time by one week of training [1]. Though these data were captured before either group participated in a structured physical fitness program during BMT, the document also stated that 49% of incoming male trainees (recruits) were unable to perform to a passing standard within the initial PFA in calendar year CY 2021, of which these data are partially representative. Such a change in CY21 due to the COVID-19 pandemic has likely influenced the outcomes recorded herein, and without accurate representations of group and individual exposures, infections, diagnoses, and specific depictions of the effects of disparate social distancing measures occurring across these candidates’ homes of record, it is impossible for the authors to fully determine and control for these impacts.

## Conclusions

Those recruits who entered DAF BMT in the period of FY19-FY23 and intended entrance into AFPSECWAR displayed greater physical fitness, were heavier, and presented greater BMI than those DAF recruits who did not seek SW entry. Variations in physical fitness were observed between the SW and non-SW cohorts across the years. Though no direct causative factors have been identified, the authors speculate that COVID-19 social distancing and organized sports regulations may have impacted the observation period, specifically FY21-FY22. Future inquiries into this population should seek to better quality specific physical training history through questionnaires and to further qualify the effects of pre-BMT physical fitness and sports history. Finally, due to the known socioecological effects of the COVID-19 pandemic, military researchers may find value in establishing protocols to identify individual recruit exposure or infection history, local social distancing protocols/safeguards, and overall physical activity profiles, independent of organized sport or structured physical fitness training in these cohorts who originate from such diverse geographical backgrounds.
